# Gap junctions amplify TRPV4 activation-initiated cell injury via modification of intracellular Ca^2+^ and Ca^2+^-dependent regulation of TXNIP

**DOI:** 10.1080/19336950.2020.1803552

**Published:** 2020-08-04

**Authors:** Xiling Zhang, Zhimin Mao, Yanru Huang, Zhen Zhang, Jian Yao

**Affiliations:** a Department of Urology, The Fourth Affiliated Hospital of China Medical University, Shenyang, China; b Division of Molecular Signaling, Department of the Advanced Biomedical Research, Interdisciplinary Graduate School of Medicine, University of Yamanashi, Chuo, Japan

**Keywords:** TRPV4, connexin43, TXNIP, oxidative stress, cell injury

## Abstract

The elevated intracellular Ca^2+^ and oxidative stress are well-reported mechanisms behind renal tubular epithelial injury initiated by various insults. Given that TRPV4 and connexin43 (Cx43) channels are activated by a wide range of stimuli and regulate both intracellular Ca^2+^ and redox status, we speculated an involvement of these channels in renal tubular cell injury. Here, we tested this possibility and explored the potential underlying mechanisms. Our results demonstrated that exposure of renal tubular epithelial cells to aminoglycoside G418 led to cell death, which was attenuated by both TRPV4 and gap junction (Gj) inhibitor. Activation of TRPV4 caused cell damage, which was associated with an early increase in Cx43 expression and function. Inhibition of Cx43 with chemical inhibitor or siRNA largely prevented TRPV4 activation-induced cell damage. Further analysis revealed that TRPV4 agonists elicited a rise in intracellular Ca^2+^ and caused a Ca^2+^-dependent elevation in TXNIP (a negative regulator of the antioxidant thioredoxin). In the presence of Gj inhibitor, however, these effects of TRPV4 were largely prevented. The depletion of intracellular Ca^2+^ with Ca^2+^ chelator BAPTA-AM or downregulation of TXNIP with siRNA significantly alleviated TRPV4 activation-initiated cell injury. Collectively, our results point to a critical involvement of TRPV4/Cx43 channel interaction in renal tubular cell injury through mechanisms involving a synergetic induction of intracellular Ca^2+^ and oxidative stress. Channel interactions could be an important mechanism underlying cell injury. Targeting channels could have therapeutic potential for the treatment of acute tubular cell injury.

## Introduction

The kidney is a vital organ that excretes metabolic wastes and maintains homeostasis of body-fluid. Because of its special structure and function, it is vulnerable to many insults, including nephrotoxic drugs. In fact, drug-initiated acute kidney injury is one of the main causes of mortality [1]. Therefore, it is necessary to clarify the underlying mechanisms and search for effective treatment for renal cell injury.

Membrane channels play important roles in the maintenance of cellular homeostasis. It is especially true for renal tubular cells because of their roles in reabsorption and excretion. Dysfunction of channels causes many disorders in renal tubular cells, including cell injury [2]. Numerous studies have demonstrated the involvement of channels in renal tubular injury. These channels include potassium channels [3], store-operated Ca^2+^ channels [4], TRP (transient receptor potential) channels [5], and gap junctions (Gjs) [6,7]. The mechanisms involved are related to channel-mediated regulation on intracellular Ca^2+^ and oxidative status [5–11].

TRPV4 is a Ca^2+^-permeable cation channel, which can be activated by many stimuli, like 4α-phorbol esters, heat, mechanical stimulation, cell swelling, and so on. In many cells, the TRPV4 channel was expressed and mediates many pathophysiological responses [9,12,13]. TRP channels also act as chemosensors that detect and mediate cell responses to toxicants [14]. Sustained activation of TRPV4 induces oxidative cell injury in multiple cell types [8,9]. Recently, TRPV4 channels have been documented to be implicated in aminoglycoside-elicited cell injury [15,16]. In this context, TRPV4 could also be critically involved in renal tubular cell injury.

Gjs, composed of connexin (Cx), are intercellular channels that allow intercellular exchange of small molecules among adjacent cells. Many essential molecules, such as Ca^2+^, ATP, and GSH, are known to be able to move through Gjs freely. GJ-mediated intercellular communication has been demonstrated to be indispensable for normal cell functions. It controls many cellular behaviors, including cell proliferation, migration, differentiation, and survival [17,18].

Channel interaction has emerged as an important mechanism mediating a variety of cell responses. Several considerations prompted us to speculate that Gjs and TRPV4 channels may cooperatively contribute to the cell injury. First, most of the biologic actions of TRPV4 are mediated by the elevation of intracellular Ca^2+^ [13], whereas Ca^2+^ signal can be transmitted, propagated, and potentiated by Gjs [17,18]; Second, TRPV4 activation results in an oxidative cell injury [8,9,19]. Interestingly, Gjs have been established as a determinant factor governing cell responses to oxidative stress [6,7,20–23]. Third, both channels are similarly activated after exposing to various stimuli and take part in the regulation of multiple cellular processes [13,18]. Furthermore, both Gjs and TRPV4 are documented to be involved in aminoglycoside-induced cell injury [7,15,16]. Lastly, it is reported that TRPV4 activation induces ATP release from Cx43 hemichannels [24]. Therefore, a cooperative regulation of cell injury by Gj and TRPV4 channels is highly possible. Here we tested this hypothesis and explored the potential interactive mechanism.

Here we presented our finding that Cx43 cooperated with TRPV4 to mediate aminoglycoside-induced renal tubular cell injury via mechanisms involving synergistic induction of intracellular Ca^2+^ and oxidative stress. Channel interaction could be an important mechanism underlying cell injury and could be targeted for therapeutic purposes.

## Materials and methods

### Reagents

TXNIP, anti-GAPDH, horseradish peroxidase–conjugated anti-rabbit IgG and phospho-p38 mitogen-activated protein kinase (MAPK) (Thr180/Tyr182) antibodies were obtained from Cell Signaling Technology (Danvers, MA, USA). Lindane was purchased from Wako, Japan. Ca^2+^ indicator Fura-2-acetoxymethyl ester was bought from Molecular Probes (Eugene, OR). 4α-Phorbol 12,13-didecanoate (4α-PDD), GSK1016790A, RN-1734, fetal bovine serum (FBS), trypsin/EDTA, antibiotics, and all other chemicals were from Sigma (Tokyo, Japan).

Lindane and BAPTA-AM were dissolved in DMSO; 4α-PPD, GSK1016790A, and RN-1734 were made in a mixture of DMSO and ethanol (1:1); G418 was dissolved in DW. These solutions were made at the concentration of at least 500-fold of their end concentration used for cell stimulation, aliquoted and stored at −20°C.

### Cell culture

The NRK-52E was bought from American Type Culture Collection (Manassas, VA, USA). For maintenance and expansion, cells were cultured in DMEM/Ham’s F-12 supplemented with 5% FBS. For experiments, cells were seeded into appropriate culture plates in the same culture media containing 0.5% FBS.

### Western blot analysis

Proteins extraction and western blot analysis were carried out as described previously [7,9]. Briefly, cellular proteins were separated by SDS-PAGE and transferred onto polyvinylidene difluoride membranes. After blocking the nonspecific binding with 5% nonfat dry milk or 3% BSA for 1 h, the membranes were reacted with the first antibodies overnight at 4°C, which was followed by incubation with the second horseradish peroxidase-conjugated anti-rabbit or anti-mouse IgG antibody. The bands in the membranes were visualized using Chemi-Lumi One L (Nacalai Tesque, Kyoto, Japan) and captured with a Fujifilm luminescent image LAS-1000 analyzer (Fujifilm, Tokyo, Japan).

### Lactate dehydrogenase (LDH) assay

Cell death was detected with lactate dehydrogenase (LDH) assay kit from Takara (LDH Cytotoxicity Detection Kit, TaKaRa), as reported previously [9].

### Live/dead cell staining

Cellstain Double Staining Kit (Dojindo, Tokyo, Japan) was used to test cell viability as described previously [9]. Briefly, seeded cells were stimulated according to specific conditions. Afterward, propidium (4 μM) [12] and calcein-AM (2 μM) were used to stain cells. Ten minutes later, the live and dead cells were discriminated. The living cells hydrolyze calcein-AM by intracellular esterase, generating green fluorescence. In contrast, the dead cells showing red fluorescence. The cell images were recorded with an Olympus CCD camera attached to the immunofluorescent microscope (IX71S1 F-2; Olympus, Tokyo) under the identical settings.

### Fluorescence cell staining

The immunofluorescent staining of Cx43 was done as previously reported [7]. Briefly, NRK-E52 cells grown in the chamber slide wells and paraformaldehyde (3%) was used to fix cells for 10 min, followed by incubation with Triton X-100 (0.5%) for an additional 15 min to permeabilize the cell membrane. After washing twice with PBS, cells were incubated with an anti-Cx43 (1:100, c6219, sigma) antibody at 4°C overnight. After washing, a fluorochrome-conjugated secondary antibody (1:200, CST) was added and allowed to react with cells at 37°C for another 2 h. Lastly, the slides were covered with Tris-buffered mowiol (pH 8.6). Cell morphology and staining were observed and photographed with a CCD camera.

### Scrape loading dye transfer (SLDT)

The gap junctional intercellular communication (GJIC) was detected using the SLDT assay [7,25]. Cells were stimulated with different stimuli, followed by the addition of lucifer yellow (LY, 0.05%). After 3 min, the background fluorescence was washed out with culture medium and then the cells were fixed with paraformaldehyde (3%). Finally, the dye transfer results were examined and photographed using a CCD camera attached to the microscope.

### Assessment of protein oxidation

OxyBlot Protein Oxidation Detection Kit (EMD Millipore, Billerica, MA) was used to evaluate the protein carbonylation [6,9,25]. Protein samples at the amount of 15 to 20 μg were denatured and derivatived with 12% SDS and 1× DNPH (2,4-dinitrophenylhydrazine), respectively. After neutralization, the samples were subjected to Western blot analysis.

### Measurement of intracellular Ca^2+^

We determined the level of intracellular Ca^2+^ as previously [9,25,26]. Briefly, cells grown in the special glass chamber wells. After washing with a balanced salt solution, cells were co-cultured with fluorescent Ca^2+^ indicator Fura-2-acetoxymethyl ester (5 μM) for 45 min. Afterward, cells were pre-treated with lindane (100 μM) for 30 min before stimulating with 4α-PDD or GSK1016790A. The F340/F380 ratio was used to calculate the level of intracellular calcium.

### siRNA transfection

siRNA transfection was performed using Hiperfect transfection reagent (Qiagen, Japan), as we have previously reported [6,7]. Briefly, NRK-E52 cells were transfected with 20 nM control, Cx43 or TXNIP siRNA. After 24 h, cells were collected and reseeded into a 12-well plate. After an additional 24-h culture, cells were stimulated with different agents as described. The supernatants were harvested for the determination of LDH release. The cellular protein was used to evaluate the evaluation of transfection efficacy.

### Statistical analysis

Microsoft Excel (Microsoft, Redmond, WA) was used for data processing. The values were expressed as mean ± SE. Student’s t-test was done to compare the value of two groups. One-way analysis of variance (ANOVA) followed by Dunnett’s test was used to compare the value of multiple groups. Statistical significance was considered at a level of 0.05 (*P*< 0.05).

## Results

### Inhibition of TRPV4 and Cx43 channels attenuates aminoglycosides-induced renal tubular cell injury

**Figure 1.**
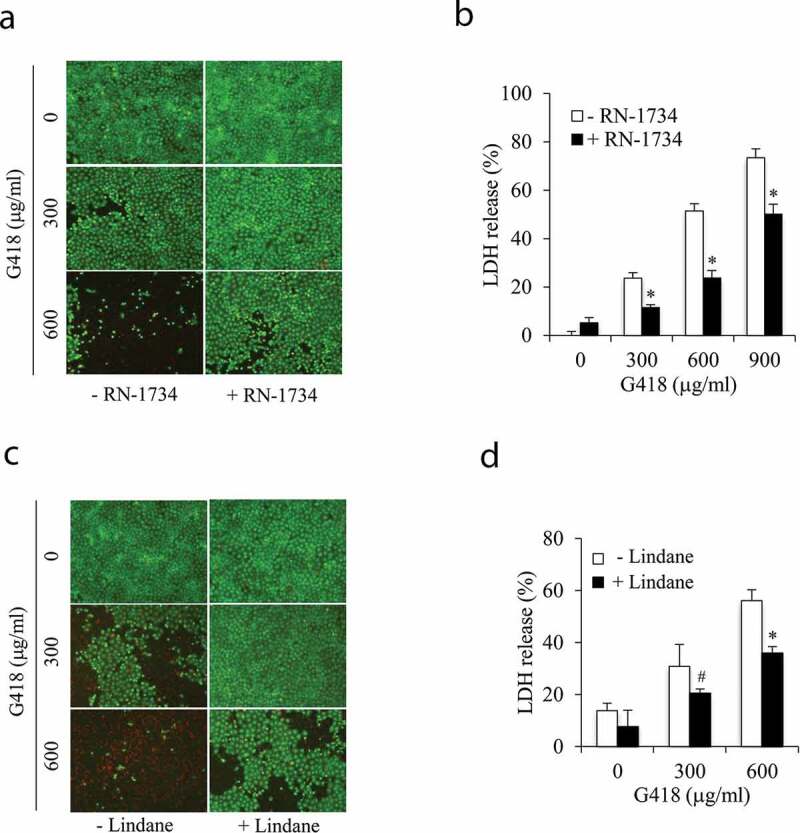
Inhibition of TRPV4 and Gj channels attenuates aminoglycoside-induced cell injury. NRK-E52 cells were pretreated with 10 μM RN-1734 or 100 μM lindane for 30 min, followed by exposing to the indicated concentrations of G418 for 48 h. Cells were subjected to calcein/PI staining (a, c) and the supernatant was collected for LDH assay (b, d). Data are expressed as mean ± SE (n = 5). *P < 0.01, ^#^P < 0.05 versus respective control without inhibitor treatment (one-way ANOVA followed by Dunnett’s test).

TRPV4 channels were involved in cell injury under many pathological situations [8,9,19]. Here we detected the potential role of TRPV4 in aminoglycoside-initiated cell death. Figure 1a and b show that exposure of NRK-E52 cells to G418, a structural analog of aminoglycoside gentamycin, resulted in cell death. The PI-positive red dead cells were markedly increased, whereas the calcein-positive stained living cells were reduced (green, Figure 1a). Consistently, the LDH release was greatly increased (1B). In the presence of TRPV4 antagonist RN-1734 [27], however, the cell damage was significantly suppressed, indicating the involvement of TRPV4 in aminoglycoside-initiated cell injury.

Intriguingly, we have previously documented a critical involvement of Gjs in aminoglycoside-induced tubular cell injury [7]. As confirmed in the current experimental setting, inhibition of Gjs with chemical inhibitor lindane markedly alleviated G418-elicited cell injury. Collectively, these observations indicate that both TRPV4 and Gj channels are implicated in renal cell injury.

### Gjs participate in TRPV4 agonist-initiated renal tubular cell injury

**Figure 2.**
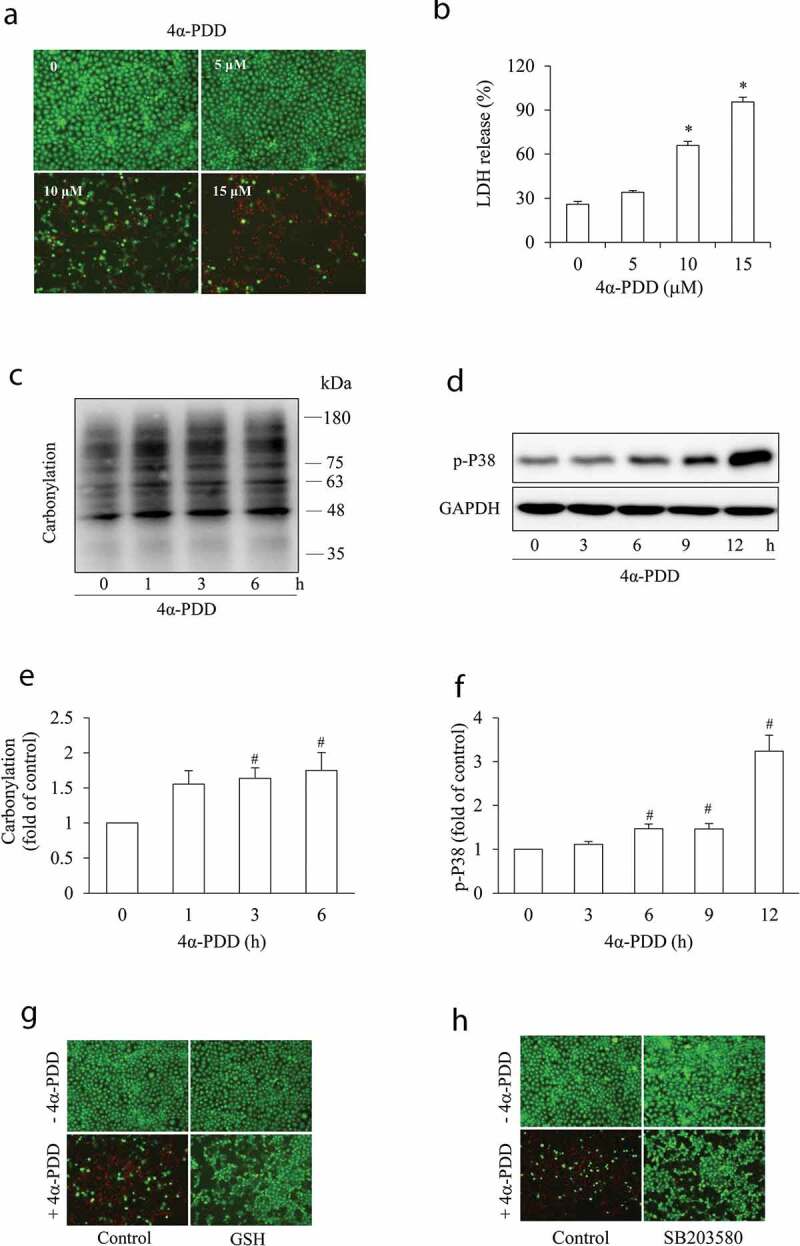
TRPV4 activation causes oxidative cell injury. (a-b) Effect of the TRPV4 agonist on cell injury. NRK-E52 cells were exposed to the indicated concentrations of 4α-PDD for 12 h. Cell viability was evaluated through calcein/PI staining (a) and LDH release (b). Data in (b) are mean ± SE (n = 5). *P < 0.01 versus zero control (one-way ANOVA followed by Dunnett’s test). (c-d) Effect of TRPV4 activation on protein carbonylation formation and p38 activation. NRK-E52 cells were exposed to 10 μM 4α-PDD for the indicated time. Cellular lysates were analyzed for carbonyl formation (c) p38 phosphorylation (d). The intensities of carbonylation signals in (c) and p-P38 signals in (d) were measured using ImageJ software and are expressed as fold of control (e, f). Results are mean ± SE (n = 3). ^#^P < 0.05 versus respective control (one-way ANOVA followed by Dunnett’s test). (g-h) Effect of antioxidant and p38 inhibitor on TRPV4 agonist-initiated cell injury. NRK-E52 cells were pretreated with 5 mM GSH (g), or 20 μM P38 SB203580 (h). Afterward, the cells were exposed to 10 μM 4α-PDD for 12 h. Cells were stained with a mixture of PI and calcein solution as described in the section of Methods. Note the obvious loss of calcein-positive (green) living cells following TRPV4 activation and its prevention by GSH and SB203580.

We then explored the relationship between TRPV4 and Gjs in the mediation of renal cell death. For this purpose, we first determined whether TRPV4 activation itself induced renal tubular cell injury. Figure 2 shows that activation of TRPV4 with a relatively high concentration of 4α-PDD, a TRPV4 agonist, induced NRK-E52 cell injury (Figure 2a and b), which was associated with increased protein carbonylation and p38 activation (Figure 2c–f). Inhibition of oxidative stress with antioxidant GSH or blockade of oxidative sensitive P38 kinase with SB203580 largely prevented cell damage (Figure 2g and h). These observations show that TRPV4 activation results in oxidative renal tubular epithelial cell death.

**Figure 3.**
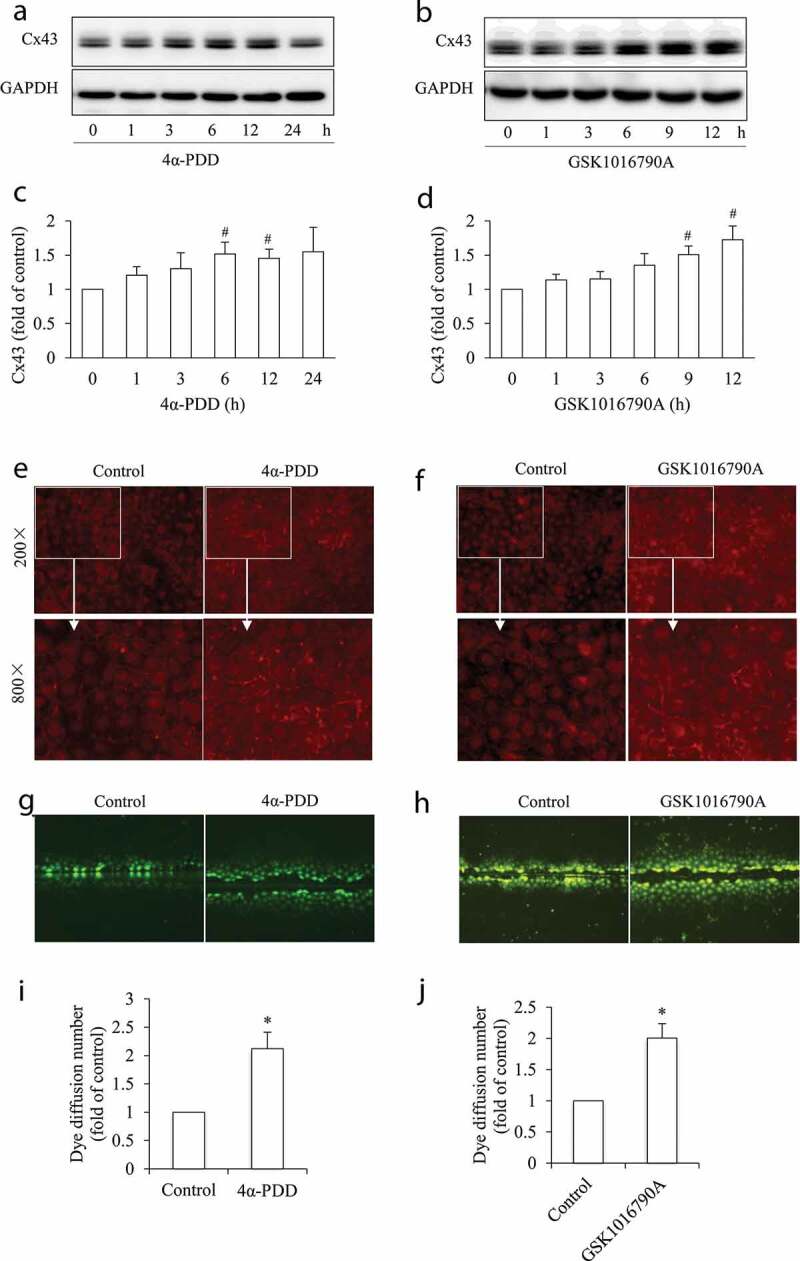
Activation of TRPV4 increases Cx43 expression and function. (a-b) Effect of TRPV4 activation on Cx43 expression. The NRK-E52 cells were exposed to 5 μM 4α-PDD (a) or 1 μM GSK1016790A (b) for the indicated times. The cellular proteins were extracted and subjected to Western blot analysis for Cx43. The intensities of Cx43 signals in (a) and (b) were measured using ImageJ software and are expressed as fold of control (c, d). Results are mean ± SE (n = 3). ^#^P < 0.05 versus respective control (one-way ANOVA followed by Dunnett’s test). (e-f) Immunofluorescent staining of Cx43. NRK-E52 cells were either left untreated or stimulated with 2.5 μM 4α-PDD (e) or 1 μM GSK1016790A (f) for 12 h and subjected to immunofluorescent staining of Cx43. Note the obviously increased fluorescence at the cell-cell contact region after TRPV4 activation. Magnification: ×200. Lower part: boxed areas are magnified (800×), (g-h) Effect of TRPV4 activation on GJIC. NRK-E52 cells were treated the same as above. The micrographs of LY diffusion into the cellular monolayer (green) after scrape loading were shown. (i-j) The dye-coupled cells in E and F were counted, and the results are expressed as fold of diffusion relative to control (mean ± SE, n = 10). *P < 0.01 versus control (student’s t-test, the same applies below).

We then tested the possible role of Cx43 channels in TRPV4 agonist-induced cell injury. As shown in Figure 3, activation of TRPV4 with 4α-PDD or GSK1016790A caused an elevated Cx43 (Figure 3a and b). Consistently, IF staining showed an enhanced Cx43 puncture at cell membrane (Figure 3e and f). Functional analysis using SLDT assay demonstrated an enhanced GJIC (Figure 3g–j). Collectively, these observations point to an increased Cx43 expression and function after TRPV4 activation.

**Figure 4.**
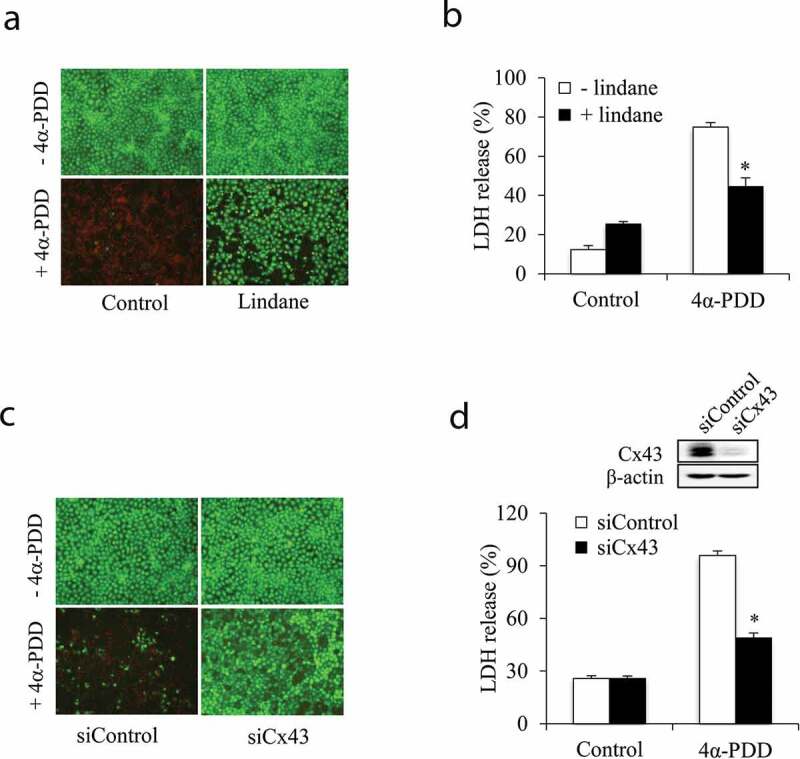
Inhibition of Gjs attenuates TRPV4 antagonist-elicited cell injury. (a, b) Effect of Gj inhibitor. NRK-E52 cells were pretreated with 100 μM lindane for 30 min. Afterward, the cells were exposed to 10 μM 4α-PDD. (c-d) Effect of Cx43 siRNA. NRK-E52 cells were pretreated with either control or Cx43 siRNA for 48 h, followed by incubation with 10 μM 4α-PDD for an additional 12 h. Results in (b) and (d) are mean ± SE (n = 5). *P < 0.01 versus respective 4α-PDD control.

To examine the potential participation of Gjs in TRPV4-initiated cell injury, we detected cell response to TRPV4 agonist in the presence of Gj inhibitor or after downregulation of Cx43, the primary functional Cx isoform in NRK-E52 cells [7], with siRNA. Figure 4 shows that these treatments significantly decreased 4α-PDD-induced cell damage, as shown by the reduced PI-positive dead cells and LDH release. These results demonstrate an important role of Cx43 channels in TRPV4-caused renal tubular cell injury.

### Induction of intracellular Ca^2+^ and Ca^2+^-dependent upregulation of TXNIP contribute to TRPV4-induced cell injury

**Figure 5.**
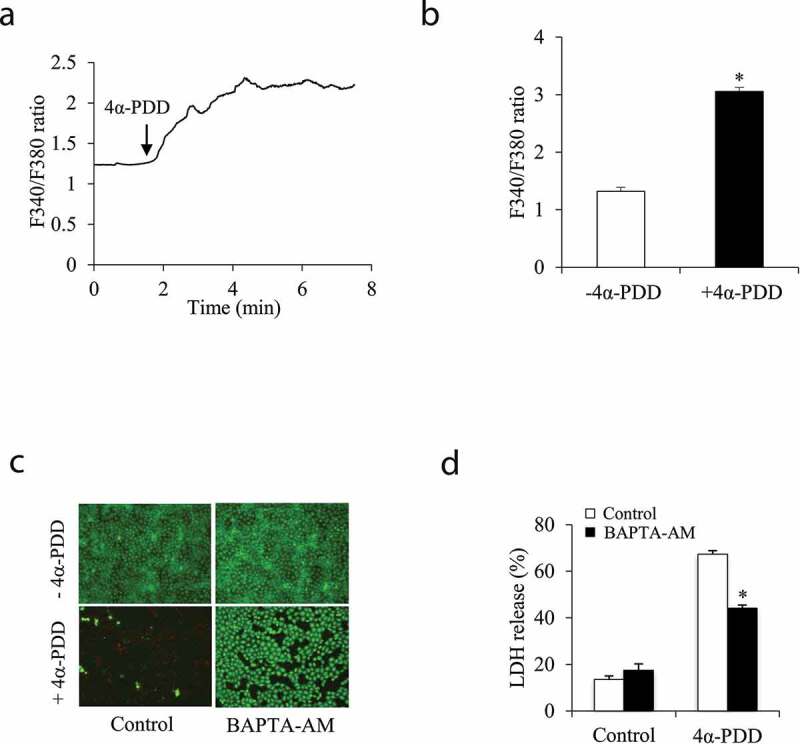
The elevated intracellular Ca^2+^ underlies TRPV4-elicited cell injury. (a, b) Effect of TRPV4 agonist on intracellular Ca^2+^. NRK-E52 cells were preloaded with Ca^2+^ indicator Fura-2. The changes in [Ca^2+^] before and after the addition of 3 μM 4α-PDD were determined through ratiometric imaging with Fura-2 at 340 nm and 380 nm (F340/F380). The arrow in (a) indicates the time of addition. The result shown in (A) is the dynamic change of intracellular Ca^2+^ among 100 cells on average before and after TRPV4 activation. (b) The intracellular Ca^2+^ level at the basal point before and the peak point after 4α-PDD addition in (A) was quantitated. Data are expressed as mean ± S.E. of three independent experiments (n = 3). *P < 0.01 versus control. (c, d) Cells were either pretreated with 10 μM BAPTA-AM or left untreated for 30 minutes before exposing to 10 μM 4α-PDD. Data in (d) are expressed as mean ± SE (n = 5). *P < 0.01 versus respective control.

It is reported that TRPV4 activation causes a rise in intracellular Ca^2+^ and Ca^2+^-mediated cell responses [9,28–30]. In this study, we also confirmed a rapid and sustained elevation in intracellular Ca^2+^ following cell exposure to 4α-PDD (Figure 5a and b). To test the role of the increased Ca^2+^ in cell injury, we depleted intracellular Ca^2+^ with Ca^2+^ chelator BAPTA-AM and determined its influence on the cell injury. Figure 5c and d show that treatment of cells with BAPTA-AM significantly attenuated the cell injury, suggesting the involvement of Ca^2+^ in the induction of cell death.

**Figure 6.**
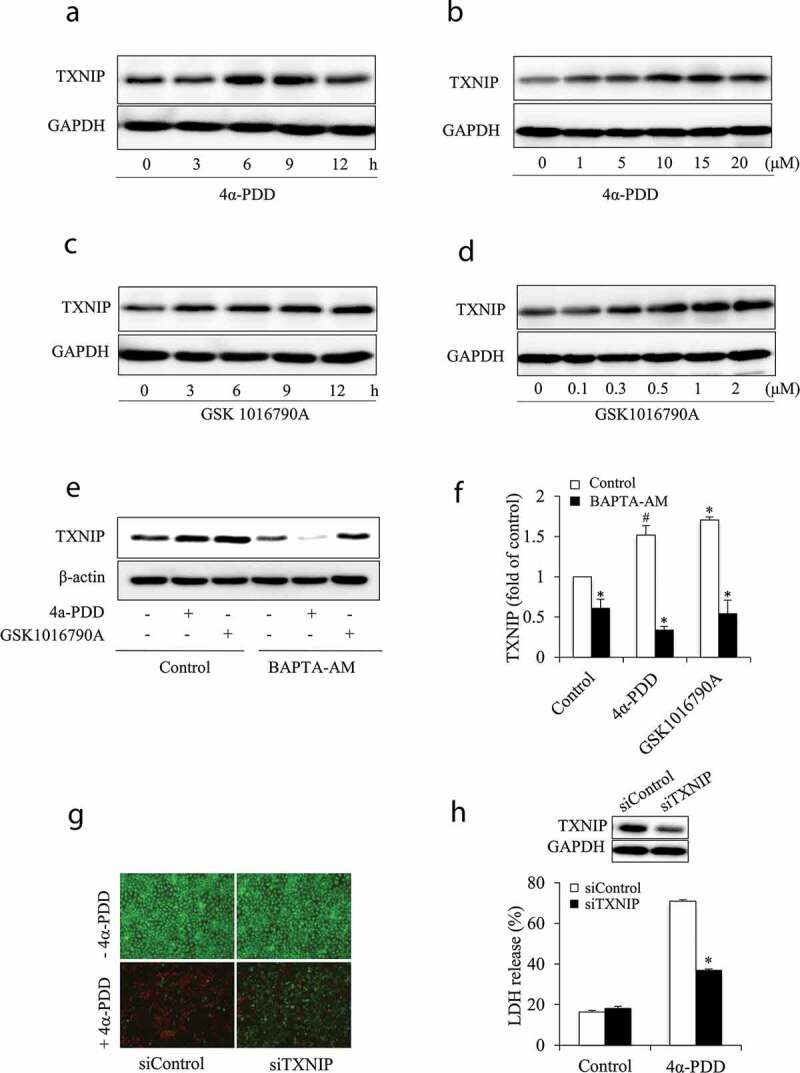
TRPV4 elicited-cell injury involves Ca^2+^-dependent induction of TXNIP. (a-d) Effect of TRPV4 agonists on TXNIP expression. NRK-E52 cells were either exposed to 5 μM 4α-PDD (a) or 1 μM GSK1016790A (c) for the indicated times, or different concentrations of 4α-PDD (b) or GSK1016790A (d) for 12 h. The cellular proteins were extracted and subjected to Western blot analysis for TXNIP. (e-f) Effect of intracellular Ca^2+^ on TRPV4-induced TXNIP. NRK-E52 cells were either pretreated with 10 μM BAPTA-AM or left untreated for 30 minutes before exposing to 10 μM 4α-PDD or 1 μM GSK1016790A for 12 h (e). The intensities of TXNIP signals in (E) were measured using ImageJ software and are expressed as fold of control (f). Results are mean ± SE (n = 3). *P < 0.01, ^#^P < 0.05 versus control. (g, h) Downregulation of TXNIP with siRNA on 4α-PDD-induced cell injury. NRK-E52 cells were treated with control or TXNIP siRNA for 48 h, followed by exposing to 10 μM 4α-PDD for an additional 12 h. Data shown are mean ± SE (n = 5). *P < 0.01 versus respective control.

Because increased Ca^2+^ has been shown to be related to ROS generation and TXNIP expression [10,31–36], we, therefore, focused on TXNIP, an endogenous thioredoxin inhibitor [32–36]. Incubation of cells with TRPV4 agonists, 4α-PDD, or GSK1016790A, resulted in an elevation in TXNIP level in a concentration- or time-dependent manner (Figure 6a–d). Furthermore, TRPV4 activation-induced TXNIP expression was reduced after depletion of intracellular Ca^2+^ with BAPTA-AM (Figure 6e and f), indicative of a mediating role of Ca^2+^. Moreover, the elevated TXNIP contributed to TRPV4 agonist-induced cell injury, as evidenced by the reduced cytotoxicity of 4α-PDD after downregulation of TXNIP with siRNA (Figure 6g and h). These observations together indicate the involvement of Ca^2+^ and TXNIP in TRPV4 activation-induced cell injury.

### Cx43 channels contribute to TRPV4 agonist-induced elevation in intracellular Ca^2+^ and TXNIP

**Figure 7.**
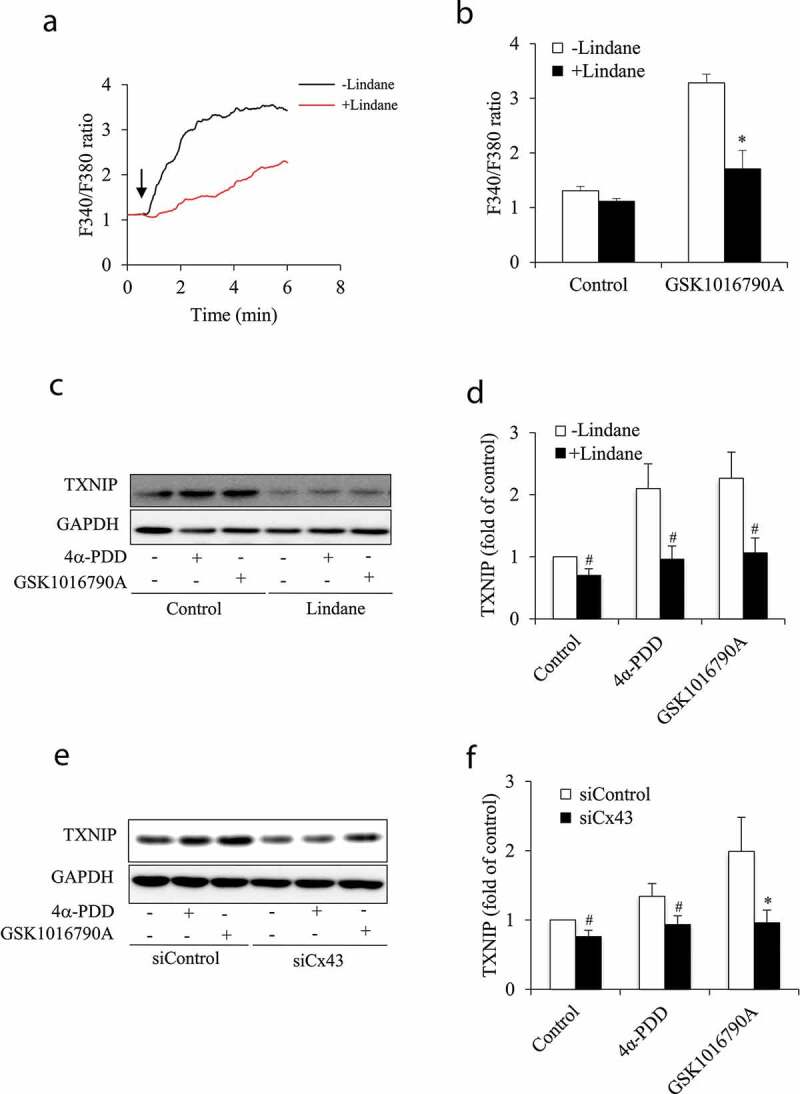
Inhibition of Cx43 blunted TRPV4 agonist-induced elevation in Ca^2+^ and TXNIP. (a) Effect of Gj inhibitor on 4α-PDD-elicited dynamic change in intracellular Ca^2+^. NRK-52E cells were pre-treated with or without 100 μM lindane for 30 min before exposing to 5 nM GSK1016790A for the indicated time. The average levels of intracellular Ca^2+^ among 100 cells were determined through ratiometric imaging of Fura-2 at 340 nm and 380 nm (F340/F380). (b) The intracellular Ca^2+^ level at basal and peak point in (a) was quantitated. Data are expressed as mean ± S.E. of three independent experiments (n = 3). *P < 0.01 versus control. (c, d) Effect of Gj inhibitor or Cx43 siRNA on TXNIP level. Cells were treated with 10 μM 4α-PDD or 1 μM GSK1016790A in the presence or absence of 100 μM lindane for 12 h (c, d). (e, f) NRK-E52 cells were treated with control or Cx43 siRNA for 48 h before exposing to TRPV4 agonists for 12 h. Cellular protein was extracted and subjected to Western blot analysis for TXNIP. The intensities of TXNIP signals in (c) and (e) were measured using ImageJ software and are expressed as folds of control in (d) and (f), respectively. Results are mean ± SE (n = 5). *P < 0.01, ^#^P < 0.05 versus respective control.

To elucidate the mechanisms by which Cx43 potentiated TRPV4-induced cell damage, we focused on the effects of Cx43 on the TRPV4-induced elevation in Ca^2+^ and TXNIP. Figure 7a and b show that Gj inhibitor lindane significantly blunted the rise of intracellular Ca^2+^ initiated by TRPV4 agonist GSK1016790A. Moreover, in agreement with the role of Ca^2+^ in the regulation of TXNIP, lindane also significantly suppressed TRPV4 agonist-triggered elevation in TXNIP (Figure 7c and d). Consistently, Cx43 siRNA also inhibited TXNIP under both basal and TRPV4 agonist-stimulated conditions. These observations thus suggest that Cx43 channels potentiate TRPV4-triggered elevation in Ca^2+^ and TXNIP.

## Discussion

In this study, we found a cooperative induction of renal tubular injury by TRPV4 and Cx43 channels through mechanisms involving modulation of intracellular Ca^2+^ and redox status. Our study suggests that channel interactions could be a significant mechanism contributing to renal tubular cell injury and that targeting channels may have therapeutic potential for the treatment of renal cell injury.

It is reported that TRPV4 can be activated by various stimuli and mediates many cell responses via Ca^2+^ influx and ATP release [13]. Prolonged activation of TRPV4, however, leads to cell damage. This phenomenon has been confirmed in several models of cell injury induced by different stimuli and is thought to be related to the elevated Ca^2+^ and oxidative stress [8,9,29]. It has been documented that TRPV4 activation led to an increased ROS level and a decreased cellular defense against oxidative stress [8]. It inhibited pro-survival PI3 K/Akt signaling pathway but activated pro-apoptotic MAP kinase P38 [9,37].

Consistent with the previous reports, we demonstrated that TRPV4 activation induced renal tubular cell injury, which was also mediated by Ca^2+^ and oxidative stress [8,9,29]. Furthermore, we characterized TXNIP as a downstream molecule mediating TRPV4-initiated oxidative stress and subsequent cell injury. As a negative regulator of the major antioxidant thioredoxin, TXNIP has a crucial effect in the control of intracellular redox status. Up-regulation of TXNIP generates ROS and results in cell death [38]. On the contrary, downregulation of TXNIP enhances cell resistance to oxidative cell injury [39]. Several previous studies have shown that TXNIP is under the control of Ca^2+^ [32–34]. Indeed, upregulation of TXNIP by TRPV4 agonists was also abolished by depletion of intracellular Ca^2+^ in this study. Thus, Ca^2+^-dependent regulation of TXNIP could be attributable to TRPV4 activation-caused cell injury.

Recently, it has been recognized that some of the actions of TRPV4 are mediated through the activation of other membrane channels. For example, TRPV4–ANO1 interaction has been documented as a pain-enhancing mechanism in the peripheral nervous system [40]. In hippocampal pyramidal neurons, activation of TRPV4 elicited a voltage-gated sodium channel through the PKA signaling pathway [41]. TRPV4 also cooperated with TRPC1/6 to mediate mechanical hyperalgesia and nociceptor sensitization [42]. In this context, it is also possible that TRPV4 induced cell injury through interaction with other membrane channels.

In this study, we found that activation of TRPV4 with different agonists led to an elevation in Cx43 expression and enhanced intercellular communication. Consistently, suppression of Cx43 significantly alleviated the cytotoxicity of TRPV4 activation in renal cells. These results showed that the action of TRPV4 activation on cell injury was, at least partially, mediated through transactivation of the Cx43 channel.

How Cx43 regulated TRPV4-induced cell damage remains unclear. The mechanism could be multiple, but the major one could be related to its regulation on intracellular Ca^2+^. As an intercellular channel, Gj transmits and propagates Ca^2+^ signals among neighboring cells [17]. In addition, hemichannel activation due to the elevated Ca^2+^ [43] may amplify and sustain TRPV4-induced Ca^2+^ signal via the purinergic signaling pathway [44,45]. In fact, activation of Cx43 hemichannels and induction of ATP release by TRPV4 has been previously reported [24]. These effects of Gjs explained why inhibition of Gjs blunted TRPV4 agonist-elicited Ca^2+^ signal. Conceivably, the other Ca^2+^-dependent cellular events, such as the elevated TXNIP and subsequent oxidative cell injury, should also be alleviated by Gj inhibition. Our results were consistent with this notion. Apart from its action on Ca^2+^, other reported mechanisms such as direct intercellular transmission and propagation of small toxic molecules, the loss of the essential molecules via hemichannels [22,23], and communication-independent regulation of cell structure and function [46] could also be involved. These mechanisms together contributed to the observed cooperative and synergistic action of Cx43 on TRPV4 activation-initiated cell injury.

Of note, other than TRPV4 channels as revealed in this study, Cx43 channels also cooperated with several other channels in the regulation of cell homeostasis [47]. As an example, a direct interaction between Cx43 and aquaporin 4 (AQP4) coordinates the intercellular movement of water and ions between astrocytes [48]. Thus, channel interaction takes part in the control of many cellular processes.

Our study is likely to be of great significance. First, our research demonstrated that TRPV4/Cx43 channel interaction could be a novel mechanism behind cell injury. The intervention of the interaction could be used to prevent or attenuate oxidative cell injury. Second, we characterized TXNIP as a potential molecule linking the increased Ca^2+^ to the occurrence of oxidative stress. Third, our research has deepened our understanding of the function of membrane channels in cell injury. Our study indicates that, although different channels could have unique roles, they may act cooperatively in the mediation of physiological and pathological responses. Nevertheless, our study also has a limitation. All the findings were obtained from *in vitro* experiments. It remains to be tested whether the same phenomenon could be validated *in vivo*. This will be the direction of our future investigation.

Taken together, our study indicates that TRPV4/Cx43 channel interaction contributes to cell injury, possibly through cooperative regulation of intracellular Ca^2+^ and intracellular redox status. Channel interaction could be a significant mechanism behind oxidative cell injury under various pathological situations.

## Supplementary Material

Supplemental Material
