# Supplementary material for: Gap junctions amplify TRPV4 activation-initiated cell injury via modification of intracellular Ca2+ and Ca2+-dependent regulation of TXNIP
Source: Channels (Austin). 2020 Aug 4;14(1):246–56. doi: 10.1080/19336950.2020.1803552 (PMC7515575; doi:10.1080/19336950.2020.1803552)
Supplement: Supplemental Material [file KCHL_A_1803552_SM8669.pdf]

Supplementary Figure for reviewer

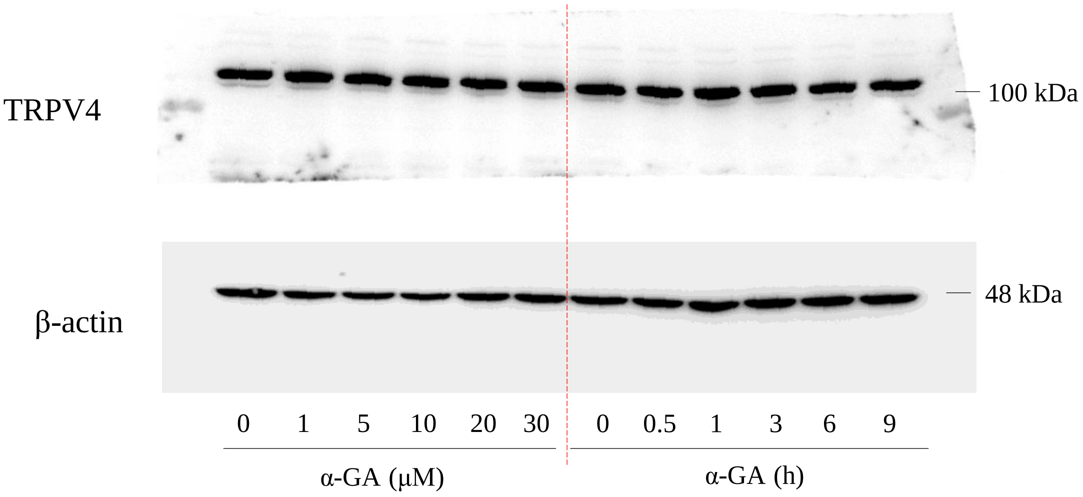

Supplementary Figure for reviewer. Effect of gap junction inhibitor α-GA (μM) on the protein level of TRPV4 in NRK cells. Cells are exposed to the indicated concentrations of α-GA for 3 h (first 6 lanes) or 10 μM α-GA for the indicated time period (last 6 lanes). The cellular proteins were collected and assayed for the protein level of TRPV4 with Western blot analysis. β-actin was used as the internal loading control. Please note the protein expression of TRPV4 in NRK cells and that its level was not altered by gap junction inhibitor a-GA.
